# Attitudes of Adolescents Toward Addictive Substances: Hope and Self-Control as Protective Factors

**DOI:** 10.3390/children12010106

**Published:** 2025-01-17

**Authors:** Serkan Cengiz, Mehmet Emin Turan, Eyüp Çelik

**Affiliations:** 1 Department of Psychology, Faculty of Arts and Sciences, Ağrı İbrahim Çeçen University, Ağrı 04100, Türkiye; scengiz@agri.edu.tr (S.C.); meturan@agri.edu.tr (M.E.T.); 2 Department of Psychological Counseling and Guidance, Faculty of Education, Sakarya University, Sakarya 54050, Türkiye

**Keywords:** attitudes toward addictive substances, ostracism, self-control, hope, adolescents

## Abstract

**Background**: Experiences of ostracism may be related to attitudes toward substance abuse. However, the protective factors underlying this relationship are still unclear. Therefore, based on the Need-threat and Self-control theory, we aimed to test a model in which self-control and hope mediate the relationship between ostracism and attitudes toward addictive substances. **Methods**: In this model, we highlighted risk factors (ostracism) and protective factors (self-control and hope). This study was a cross-sectional data analysis of 787 students (52.50% boys, 47.50% girls; M*_age_* = 15.69, SD = 1.12). **Results:** The results revealed that ostracism was positively associated with attitudes toward addictive substances, and evidence was provided that this relationship was mediated by hope and self-control. Ultimately, the research highlights the link between ostracism and attitudes toward addictive substances, identifying hope and self-control as mediators. **Conclusions**: This study highlights individual risk and protective factors related to attitudes toward addictive substances and offers new perspectives on ways to prevent and reduce adolescents’ positive attitudes toward substance use. School counselors and educators should help students strengthen skills such as hope and self-control to prevent them from developing positive attitudes toward substance use in the future.

## 1. Introduction

Substance use is among the most serious public health problems, especially during adolescence [1]. During this period, the level of curiosity is high, and young individuals are sensitive to peer pressure, showing a strong tendency to rebel against authority and low self-esteem [2]. These situations can make people vulnerable to substance use and addiction. A comprehensive study worldwide reported that approximately 4.2 million adolescents between the ages of 12 and 17 use substances [3]. Studies in Africa have shown an increasing prevalence of substance abuse among adolescents of approximately 41.6% [4]. The results from the National Survey on Drug Use and Health in the United States have shown that nonmedical use of prescription opioids has increased among adolescents aged 12–17 years [5]. A large-scale study in Europe, including 25 countries, revealed that adolescent cannabis use is increasing [6].

Substance abuse and addiction are quite common among many adolescents in Turkey, as in many other countries [7,8]. However, studies on the prevalence of its use, especially among adolescents, in Turkey are limited because of a lack of coordination between institutions, public health concerns, and cultural factors [9,10]. Substance use is a complex, multifactorial health disorder characterized by a chronic and recurrent nature, affecting almost every family globally [11]. On the other hand, previous studies have focused mostly on the consequences of substance use and attitudes toward addictive substances have not been sufficiently examined. Indeed, attitudes generally precede and influence behavior [12]. A positive attitude indicates a tendency to approach different addictive substances, whereas a negative attitude indicates a tendency to stay away from these substances. Therefore, knowing the risk factors that lead to substance use is important in terms of the early detection of young people at risk of use or the determination of interventions to reduce the risk of future substance use.

### 1.1. Ostracism and Attitudes Toward Addictive Substances

Among the many antecedents of substance abuse among adolescents, ostracism may be particularly problematic. Ostracism can be situations in which one feels emotionally and physically separate from others [13]. During adolescence, relationships with peers can play an important role in establishing healthy habits. However, compared with other developmental periods, there is a heightened sensitivity to ostracism during adolescence because of factors such as the peak importance of peer groups and the most frequent occurrence of exclusionary behaviors during this period [14].

Individuals who are excluded generally report a sense of meaninglessness, solitude, depressive symptoms, and alienation [15,16]. The perception of ostracism and the resulting threat to basic psychological needs can lead to harmful behaviors, including substance use. Many studies have revealed a significant relationship between ostracism and substance use [17,18]. For example, Levine [19] reported that ostracism poses a risk for cigarette use. One study also reported that isolation from peers increased behavioral responses to cocaine and amphetamines [20].

According to Williams’ need-threat model [21], even the simplest form of ostracism is perceived by people as threatening basic needs. In the initial stage, ostracism indicates social danger. A person’s reaction to ostracism is reflexive, and the ostracism felt is experienced as social pain. As a result, because of the long-term effects of permanent ostracism, a sudden decline in the mood of individuals may occur, and negative behavioral reactions (e.g., smoking, alcohol, drugs, addictive drug use, etc.) may be observed. The target of ostracism may be attempting to exert control to cope with negative emotions and behaviors or reconnecting with others while maintaining hope. Therefore, we included self-control and hope as potential mediators of the relationship between ostracism and attitudes toward substance abuse.

### 1.2. The Mediating Role of Hope

Another factor that may affect attitudes toward addictive substance use is the level of hope. Hope is the process of thinking about paths leading to personal goals (pathway thinking) and the ability to think in a way that motivates oneself to reach the goal via these paths (agency thinking) [22]. According to hope theory, the most hopeful individuals are those high in path and action thinking. In contrast, those with low hopes may have difficulty finding available methods to achieve their goals and may be less motivated to achieve what they want. Since greater hope is associated with greater self-confidence, well-being, coping flexibility, and emotion regulation skills [23,24], it can be considered a protective factor in preventing substance use. Gutierrez [25] reported that hope plays a protective role in recovery from withdrawal because it prevents many behavioral and psychological problems in life.

However, hope involves individual ways of thinking and personal motivation and is also affected by communicating with others and interpersonal interactions. Within the framework of the need-threat model [21], ostracism threatens a basic need to belong. This model also emphasizes that individuals respond to ostracism in three sequential stages: reflexive, reflective, and resignation. Hope is one of the characteristics that significantly influences a person’s response to ostracism at different stages of the experience of ostracism [26]. Therefore, the state of hope can be affected by ostracism in social environments and, accordingly, affect the psychological state of individuals.

Adolescents may have high levels of hope and low levels of both components. The lack of a sense of belonging and the hopelessness experienced may prevent young people from achieving a role in their social lives and, most importantly, their health identity. This may lead to substance use disorders. Therefore, we suggest that hope is likely to mediate the relationship between ostracism and attitudes toward addictive substances.

### 1.3. The Mediating Role of Self-Control

Another characteristic associated with attitudes toward addictive substances is self-control. Self-control can be defined as the capacity of a person to control or regulate their emotions, cognition, and behavior [27]. Research has shown that self-control is a strong protective factor in behavioral addiction and health risk behavior [28,29]. Individuals with greater self-control are better able to protect themselves from psychological distress when faced with a stressful situation. In contrast, individuals with low self-control are characterized as impulsive, insensitive, risk-taking, and more narrow-minded. In other words, these individuals enjoy taking risks and engaging in risky behaviors, tend to be self-centered, and have less tolerance [30].

One of the main reasons for the development of low self-control is ostracism [31]. According to the temporal need-threat model, ostracism threatens various psychological needs of individuals, including their sense of control. Considering this approach, in the reflexive stage, individuals affected by ostracism experience social pain when their needs for belonging and control are threatened. In reinforcing thwarted needs, high self-control ability may reactivate prosocial tendencies. Conversely, underdeveloped self-regulatory mechanisms may not adequately process important social stimuli, which may lead to positive attitudes toward substance use.

Gottfredson and Hirschi [32] proposed self-control theory as part of their general theory of crime, suggesting that people are motivated to engage in behaviors that increase their pleasure or decrease their pain. However, criminal and substance use behaviors depend on a person’s ability to exercise self-control when appropriate opportunities arise. Low self-control increases the likelihood of engaging in actions such as crime and substance abuse. For these individuals, using addictive substances may seem simple and exciting, especially because individuals cannot see the long-term consequences of their actions and the harm they may cause. Previous researchers have reported that individuals with lower levels of self-control are at greater risk of using alcohol, tobacco, and marijuana [33,34]. In addition to its relationship with attitudes toward addictive substances, recent research has shown that low self-control is also associated with emotional problems such as shyness, loneliness, low self-esteem, and psychological anxiety [35,36,37]. As a result, adolescents with a lack of self-control are more likely to develop positive attitudes and behaviors toward addictive substances.

### 1.4. Present Study

One of the important findings from school-based prevention research is that information dissemination interventions help change knowledge or attitudes about addictive substances [38]. However, most current prevention and information programs involve interventions designed for risk behaviors other than school dropout or substance abuse, such as sexual risk behavior, problematic internet, and media use [39,40]. The literature indicates the need for protective mechanisms that can counteract ostracism in adolescence. In addition, most studies on the relationship between ostracism and attitudes toward addictive substances have focused on adults, especially university students, rather than adolescents. Also, a better understanding of the increased sensitivity to interpersonal stressors, such as exclusion from a group, among individuals with positive attitudes toward substance use and evaluating them as protective mechanisms, such as hope and self-control, may have important implications for determining potential treatment strategies. Finally, although the relationships between the variables in the study are theoretically supported in different studies, no studies have been found that include the hypotheses in the current study. To fill these gaps, the current study used a sample of Turkish adolescents to examine the mediating effects of self-control and hope. This study aimed to test the relationships between ostracism and attitudes toward addictive substances and the underlying mechanisms in a sample of adolescents in Turkey. We formulate the research hypotheses as follows:

**H_1_.:**  *Ostracism is positively associated with attitudes toward addictive substances.*

**H_2_.:**  *Hope mediates the relationship between ostracism and attitudes toward addictive substances.*

**H_3_.:**  *Self-control mediates the relationship between ostracism and attitudes toward addictive substances.*

## 2. Materials and Methods

### 2.1. Participants

First, the schools where the application would be carried out were selected via a convenience sampling approach, considering their willingness to participate and their accessibility to the researchers. Then, the researchers established some criteria for participation in the study. (i) No history of previous use of addictive substances, (ii) no psychological treatment, (iii) not meeting the criteria for psychosis or a serious personality disorder, and (iv) parental consent to participate in the study. Finally, the G*Power 3.1 program analysis test was performed to determine the number of participants in the current study according to the criteria. The results showed a minimum sample size of 743 for models with an alpha level of 0.05, an effect size of 0.12, and a power level of 0.95. Considering possible data loss, we applied the scales to 800 students. After the exclusion of missing answers and data from 13 individuals who did not meet the normality criteria, the final sample consisted of 787 students. (52.50% females, 47.50% males; M*_age_* = 15.69, *SD* = 1.12). The demographic characteristics are shown in Table 1.

### 2.2. Measures

#### 2.2.1. Ostracism Experience Scale for Adolescents (OES-A)

OES-A [41] is an 11-item self-report measure designed to assess an individual’s perceptions of being ignored by or excluded from the social group. Each item (e.g., “In general, others treat me as if I am invisible”) on the OES-A is answered on a 5-point Likert-type scale varying between 1 (never) and 7 (always). Akın et al. [42] validated the scale in Turkish. Cronbach’s alpha coefficient for the OES-A was 0.88 in this study.

#### 2.2.2. Brief Self-Control Scale (BSCS)

The BSBC [43] includes 13 items (e.g., “I can work effectively toward long-term goals.”) that are scored on a 5-point Likert-type scale ranging from 1 (not at all like me) to 7 (very much like me). The BSBC is a self-reported measure designed to measure self-control. The BSBC was validated in Turkish by Eroğlu [44]. The Cronbach’s alpha coefficient for the BSBC was 0.66.

#### 2.2.3. Hope Scale (HS)

The HS [45] is a commonly used self-reported scale constructed to assess a respondent’s level of hope. The HS comprises 12 items (e.g., “I can think of many ways to get out of a jam”.) that are rated on an 8-point Likert-type scale ranging from 1 (definitely false) to 5 (definitely true). Turkish validation of the HS indicated satisfactory psychometric properties [46]. The Cronbach’s alpha coefficient for the HS was 0.85 in this study.

#### 2.2.4. Attitudes Toward Addictive Substances Scale (ATASS)

The ATASS [47] is a unidimensional self-reported scale developed to assess attitudes toward addictive substances in high school students. The ATASS includes 45 items (e.g., “I think I would be a happier person if I were using an addictive substance”). Each item was rated on a 5-point Likert-type scale varying between 1 (absolutely inappropriate) and 5 (absolutely appropriate). A higher score on the scale represents a greater level of negative attitudes toward addictive substances. The ATASS has shown good evidence of reliability and validity [47]. The Cronbach’s alpha coefficient for the ATASS was 0.93 in this study.

### 2.3. Procedure

The current study was approved by the research ethics committee of the university where the first author works. Informed consent was obtained from school administrators, teachers, families, and students before starting the study. The participants were informed about the nature of the study. We ensured that all the participants were volunteers. We also stated that students could withdraw at any time, even if they started answering the survey items. We told the students that their responses would remain confidential. When determining the period for administering the scales, we considered school exams, academic activities, courses, and project work. In line with this planning, the researchers were present in the classrooms during the application. It took an average of 30 min for the participants to complete all the surveys. We completed the data collection process in 60 days.

### 2.4. Data Analysis

**Figure 1:**
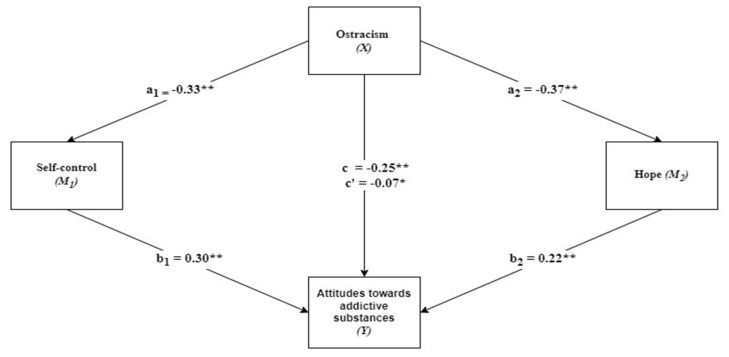
Mediation model (** *p* < 0.001; * *p* < 0.05).

In this study, multiple linear regression analysis was conducted to see the confounding factors and isolate the relationship of interest. In multiple linear regression, researchers can include many covariates at the same time. In the mediation model of the present study (see Figure 1), we propose that self-control and hope are mediators (M) of the relationship between ostracism (X) and attitudes toward addictive substances (Y). We employed bootstrapping (10,000 samples) to analyze the extent to which ostracism—attitudes toward addictive substance relationships—are mediated by self-control—hope [48]. Kappa-squared (κ2) with 95% bootstrapped confidence intervals were calculated to estimate the effect size for the indirect effect (Preacher and Kelley, 2011). Based on a rule of thumb proposed by Preacher and Kelley [49], the size of κ2, concerning Cohen’s criteria for squared correlation coefficients of 0.01, 0.09, and 0.25, refer to small, medium, and large effects, respectively. All analyses were carried out via SPSS v.26 and macro-PROCESS v4.2.

## 3. Results

### 3.1. Descriptive Results

Descriptive statistics (e.g., means, standard deviations, normality tests), bivariate correlation coefficients, and internal consistency reliability estimates for the variables of the present study are reported. The values of skewness (range = −0.72 and 0.23) and kurtosis (range = −0.71 and 0.16) fell within the “good” range of a normal distribution with a conventional frame of reference for skewness and kurtosis scores < |1| [50]. The results of the correlation analysis revealed that ostracism was significantly negatively correlated with attitudes toward addictive substances, self-control, and hope. Attitudes toward addictive substances were also significantly positively correlated with self-control and hope. There was a significant positive relationship between self-control and hope. The descriptive statistics and correlations are shown in Table 2.

### 3.2. Mediation Analysis

The results showed that ostracism had a significant positive predictive effect on self-control (β = −0.33, *p* < 0.001) and hope (β = −0.37, *p* < 0.001). Ostracism explained 11% of the variance in self-control and 14% of the variance in hope. Ostracism (β = −0.07, *p* < 0.05), self-control (β = 0.30, *p* < 0.001), and hope (β = 0.22, *p* < 0.001) had significant positive predictive effects on attitudes toward addictive substances. Collectively, these three variables explained 22% of the variance in attitudes toward addictive substances. In addition, the indirect effect of ostracism on attitudes toward addictive substances was statistically significant through self-control (effect = −0.29, 95% CI [−0.40, −0.20]) and hope (effect = −0.24, 95% CI [−0.34, −0.14]). This result shows that self-control and hope mediate the relationship between ostracism and attitudes toward addictive substances (see Table 3 and Table 4, and Figure 1).

## 4. Discussion

The current study revealed that ostracism was associated with attitudes toward substance use. These findings are consistent with other studies associated with substance use [51,52]. Many problematic substance users may have experienced significant disadvantages and ostracism. Furthermore, many studies have shown that ostracism is a risk factor for substance abuse [53,54]. Laws et al. [55] reported that individuals may turn to alcohol as a means of reducing stress due to increased arousal and the negative effects of social rejection. According to the need-threat model [21], ostracism threatens people’s need for meaningful existence and belonging. Adolescents spend a significant amount of time with their peers and are therefore highly sensitive to social experiences with them. Adolescents may be overreactive to ostracism, which is a distressing experience [56]. Overreactions and perceived stress to negative experiences such as ostracism may sensitize individuals to positive attitudes toward substance abuse. Thus, youth with increased anxiety and sensitivity to ostracism are more likely to use substances during adolescence than youth with less sensitivity. These findings provide valuable insights into the mechanism of the relationship between ostracism and substance use.

Another study revealed that self-control in adolescents significantly mediated the relationship between ostracism and attitudes toward substance use. This result provides evidence that adolescents with greater self-control skills are less likely to develop positive attitudes toward addictive substances in the face of ostracism. Compared with adults, adolescents have weaker self-control skills [57], so they may be prone to substance abuse when exposed to ostracism. Ostracism has many negative consequences, but few studies have investigated whether self-control helps explain the relationship between ostracism and attitudes toward substance use. Many studies have shown that people often have difficulty controlling impulses when exposed to ostracism [58,59]. According to Gottfredson and Hirschi’s [32] self-control theory, people with low self-control tend to be more impulsive and engage in criminal activities. The reason for this tendency is that substance abuse provides instant gratification. Therefore, these deficiencies in self-control can lead to serious problems such as substance addiction and play a serious role in relapse after a period of abstinence.

Our findings also suggest that hope moderates the effect of ostracism on attitudes toward substance use. Consistent with this result, previous research has significantly associated ostracism with hope [60,61]. In terms of the need-threat model [21], the first stage of ostracism is the immediate response stage, where people enter the reflective stage, which involves short-term cognitive or behavioral responses to cope with ostracism. In this context, hope can prevent the negative effects of ostracism in the long term by affecting a person’s sense of purpose and positive self-worth. Indeed, hope has been associated with adaptive coping and greater adaptability when faced with stress [62,63]. In addition, hope may be an important component of recovery from substance abuse and may provide willpower in quitting substance use [64]. Research has shown that hope activity and hope pathway scores contribute to drug abstinence [65]. In an experimental study, Koehn and Cutcliffe [66] demonstrated that hope is a resource that contributes positively to clients’ mobility, disposition, and activity in substance abuse counseling. In this context, a diminished sense of hope can act as a barrier to recovery in the face of many negative experiences, such as ostracism, and may lead individuals to believe that addictive substances provide them with a better quality of life. When individuals feel hopeful about their future, they are more likely to set meaningful goals and continue their efforts even when faced with setbacks. Interventions that promote hope and self-control can be used to manage ostracism. Additionally, although psychological constructs such as the relationship between ostracism and social media addiction are well documented [67,68], research on the psychological aspects of substance use [69] is limited. The present study may also shed light on the psychological aspect of substance addiction.

## 5. Limitations and Future Suggestions

This study makes several contributions to the existing research and practice in literature. This study incorporated the need-threat and self-control theory into the relationship between ostracism and attitudes toward substance use and created a new explanatory model for attitudes toward addictive substances. The results show that ostracism can predict attitudes toward substance abuse through the mediating effects of hope and self-control. These findings prove that adolescents who are excluded by peers and others exhibit less social control and hope, which increases their risk of developing deviant substance abuse. This study not only provides an explanatory framework for attitudes toward substance abuse but also contributes significantly to research on variables related to attitudes toward substance abuse.

Moreover, the results of this study provide ideas on how to address attitudes toward substance use in adolescence, especially in Turkish society. Considering the positive effect of ostracism on attitudes toward addictive substances, hope-enhancing measures should be provided by both families and educators. In addition, studies or intervention projects focused on increasing self-control should be further evaluated and implemented to prevent and address substance use among adolescents.

In addition, the findings of this study provide valuable insights for psychology counselors (especially school psychology counselors) and substance abuse specialists. The psychological constructs of hope and self-control, which are the mediating variables of this study, may be added to school-based preventive intervention studies conducted during adolescence. Studies on the management of ostracism may emphasize school-based interventions. To prevent the development of positive attitudes toward substance abuse, family- and community-based curriculum programs are also implemented in many countries. The findings of this study can also be added to family- and community-based curricula. In these programs, components such as managing ostracism and promoting hope and self-control can be added.

On the other hand, the current study has several limitations. First, since a cross-sectional design was used, it is difficult to draw causal inferences between variables. Future studies could design a controlled laboratory study to further confirm the results of the current study. Second, the sample of this study included only adolescents who continued their education in one region of Turkey, which may limit the generalizability of the results; therefore, future studies can use students from different regions of Turkey or other countries to validate the current results. Finally, the results of this study were tested with a correlational model. Future studies can test the current variables by establishing experimental models to support the results of this study.

## Figures and Tables

**Table 1:** Demographic characteristics of participants.

Variable	Level	*n*	%
Gender	Female	414	52.50
	Male	373	47.50
Grade	9th	241	30.62
	10th	232	29.48
	11th	280	35.58
	12th	34	4.32
Perceived school success	Below average	118	14.99
	Average	538	68.36
	Above average	131	16.65
Perceived socioeconomic status	Below average	127	16.14
	Average	580	73.70
	Above average	80	10.16
Age	Minimum	13	
	Maximum	18	
	Mean	15.69	
	SD	1.12	

**Table 2:** Descriptive statistics and correlations.

	Descriptive Statistics					Correlation			
Variable	M	SD	Skewness	Kurtosis	α	1	2	3	4
1. Ostracism	26.97	10.05	0.23	−0.66	0.88	—	−0.34 **	−0.37 **	−0.25 **
2. Self-control	43.80	8.30	−0.03	−0.24	0.66		—	0.39 **	0.41 **
3. Hope	46.51	12.38	−0.72	0.16	0.85			—	0.36 **
4. Attitudes towards addictive substances	180.21	29.73	−0.60	−0.71	0.93				—

Note. ** *p* < 0.01.

**Table 3:** Unstandardized coefficients of the model.

		Outcome Variable			
		*M*_1_ (Self-Control)			
Variable		Coeff.	*SE*	*t*	*p*
Constant	*i*_M1_	51.25	0.80	64.12	<0.001
*X* (Ostracism)	*a*_1_	−0.28	0.03	−9.95	<0.001
		*R*^2^ = 0.11 *F* = 98.90; *p* < 0.001			
		*M_2_* (Hope)			
Constant	*i*_M2_	58.82	1.18	50.06	<0.001
*X* (Ostracism)	*a*_2_	−0.46	0.04	−11.18	<0.001
		*R*^2^ = 0.14 *F* = 125.05; *p* < 0.001			
		*Y* (Attitudes towards addictive substances)			
Constant	*by*	115.01	7.39	15.57	<0.001
*X* (Ostracism)	*c’*	−0.21	0.10	−2.02	<0.05
*M*_1_ (Self-control)	*b*_1_	1.07	0.13	8.43	<0.001
*M*_2_ (Hope)	*b*_2_	0.52	0.09	6.04	<0.001
		*R*^2^ = 0.22 *F* = 72.02; *p* < 0.001			

Note. *SE* = standard error. Coeff = unstandardized coefficient. *X* = independent variable; *M* = mediator variable; *Y* = dependent variable.

**Table 4:** Standardized indirect effects of ostracism on attitudes towards addictive substances.

Path	Effect	*SE*	BootLLCI	BootULCI
Total indirect effect	−0.18	0.02	−0.22	−0.14
Ostracism → self-control → attitudes towards addictive substances	−0.10	0.02	−0.13	−0.07
Ostracism → hope → attitudes towards addictive substances	−0.08	0.02	−0.11	−0.05

Note. Number of bootstrap samples for percentile bootstrap confidence intervals: 10,000.

## Data Availability

The data presented in this study are available on request from the corresponding author.
